# Reducing Communication Delays and Improving Quality of Care with a Tuberculosis Laboratory Information System in Resource Poor Environments: A Cluster Randomized Controlled Trial

**DOI:** 10.1371/journal.pone.0090110

**Published:** 2014-04-10

**Authors:** Joaquín A. Blaya, Sonya S. Shin, Martin Yagui, Carmen Contreras, Peter Cegielski, Gloria Yale, Carmen Suarez, Luis Asencios, Jaime Bayona, Jihoon Kim, Hamish S. F. Fraser

**Affiliations:** 1 Division of Global Health Equity, Brigham and Women's Hospital, Boston, Massachusetts, United States of America; 2 eHealth Systems, Santiago, Chile; 3 Instituto Nacional de Salud, Lima, Peru; 4 Socios en Salud Sucursal Peru, Lima, Peru; 5 Centers for Disease Control and Prevention, Atlanta, Georgia, United States of America; 6 DISA V Lima Ciudad, Lima, Peru; 7 DISA IV Lima Este, Lima, Peru; 8 Division of Biomedical Informatics, University of California San Diego, La Jolla, California, United States of America; 9 Partners In Health, Boston, Massachusetts, United States of America; 10 University of Leeds, Leeds, United Kingdom; McGill University, Canada

## Abstract

**Background:**

Lost, delayed or incorrect laboratory results are associated with delays in initiating treatment. Delays in treatment for Multi-Drug Resistant Tuberculosis (MDR-TB) can worsen patient outcomes and increase transmission. The objective of this study was to evaluate the impact of a laboratory information system in reducing delays and the time for MDR-TB patients to culture convert (stop transmitting).

**Methods:**

Setting: 78 primary Health Centers (HCs) in Lima, Peru. Participants lived within the catchment area of participating HCs and had at least one MDR-TB risk factor. The study design was a cluster randomized controlled trial with baseline data. The intervention was the e-Chasqui web-based laboratory information system. Main outcome measures were: times to communicate a result; to start or change a patient's treatment; and for that patient to culture convert.

**Results:**

1671 patients were enrolled. Intervention HCs took significantly less time to receive drug susceptibility test (DST) (median 11 vs. 17 days, Hazard Ratio 0.67 [0.62–0.72]) and culture (5 vs. 8 days, 0.68 [0.65–0.72]) results. The time to treatment was not significantly different, but patients in intervention HCs took 16 days (20%) less time to culture convert (p = 0.047).

**Conclusions:**

The eChasqui system reduced the time to communicate results between laboratories and HCs and time to culture conversion. It is now used in over 259 HCs covering 4.1 million people. This is the first randomized controlled trial of a laboratory information system in a developing country for any disease and the only study worldwide to show clinical impact of such a system.

**Trial Registration:**

ClinicalTrials.gov NCT01201941

## Introduction

Diagnosis and management of many diseases requires prompt, reliable access to laboratory investigations. Delayed, inaccurate or lost data can put patients at risk and lead to increased costs through duplicate testing [1] and late stage management of disease. This is particularly important in infectious diseases where untreated patients can pose a risk to family and community. Studies in developed countries have shown that laboratory information systems can improve timeliness and quality of laboratory data [2] and the timeliness of clinical responses [3], [4], however, none have shown impact on patient outcomes. Health systems in developing countries with limited infrastructure are particularly vulnerable to deficiencies in all stages of laboratory testing from sending and tracking samples, to ensuring accuracy of results, to getting results back to the clinician [5].

Multi drug resistant Tuberculosis (MDR-TB) is an increasingly common, dangerous and highly infectious disease where diagnosis and management is critically dependent on laboratory testing. In 2010 the World Health Organization (WHO) and the Global Fund for AIDS, Tuberculosis and Malaria planned to expand the number of patients on drug resistant tuberculosis treatment annually from 30,000 to 450,000 by 2015. This expansion will require over US$15 billion. Effective information systems will be essential to scale these projects and ensure high quality care is delivered in an efficient way. There have been multiple calls for more evidence regarding their impact [6], especially using randomized trials [7], however, there is still little evidence of their impact worldwide and in resource-poor settings [8].

TB is a chronic infectious disease that kills almost two million people per year in the developing world. Diagnosis of MDR-TB – defined as TB caused by *Mycobacterium tuberculosis* strains resistant to at least isoniazid and rifampicin – requires a drug susceptibility test (DST) which is usually performed at a district, national or even supranational level. The emergence of extensively drug-resistant tuberculosis (XDR-TB) heightens the urgency of prompt diagnosis to curb the excessive mortality and ongoing transmission associated with highly resistant strains [9]. Prompt treatment with individualized drug regimens based on DST improves patient outcomes [10] and reduces the risk of amplification of drug resistance and ongoing transmission [11], [12].

To improve detection and treatment of MDR-TB and XDR-TB, the Peruvian Ministry of Health decentralized rapid and conventional 1^st^ line DSTs from the National Reference Laboratory (NRL) to district laboratories [13]. However, like other countries, communication of results between central and local laboratories and clinical facilities was problematic. A baseline assessment found that 10% of results took over two months to arrive [14]. To reduce these delays, we developed and implemented a laboratory information system, e-Chasqui, to communicate data between the NRL, two district laboratories and 12 HCs [15]. This system was shown to decrease the number of reporting errors to HCs by up to 87%, most importantly eliminating missing results [16].

We conducted a cluster randomized controlled trial to evaluate the effectiveness of the e-Chasqui laboratory information system in reducing communication delays, time to treatment, and time to culture conversion (the end of disease transmission) within the TB program in Peru.

## Methods

A cluster randomized controlled trial (RCT) tested the effect of the laboratory information system e-Chasqui in reducing the time to communicate patients' test results, to start them on appropriate treatment, and to achieving culture conversion. The trial is reported according to the CONSORT statement [17]. This trial was performed within a larger observational study evaluating the impact of expanded laboratory capacity in the district laboratories [14]. All data were collected prospectively. This study was approved by the Partners Healthcare Human Research Committee and the Peruvian National Institute of Health, and has been registered in ClinicalTrials.gov with identifier NCT01201941. Patient consent was not obtained because both institutional review boards waived the need for written informed consent from the participants because this was part of routine clinical care and the study was secondary use of clinical data. The protocol for this trial and supporting CONSORT checklist are available as supporting information; see Checklist S1, Protocol Secondary Use S1, and Procotol Summary S1.

### Study Settings

**Figure 1:**
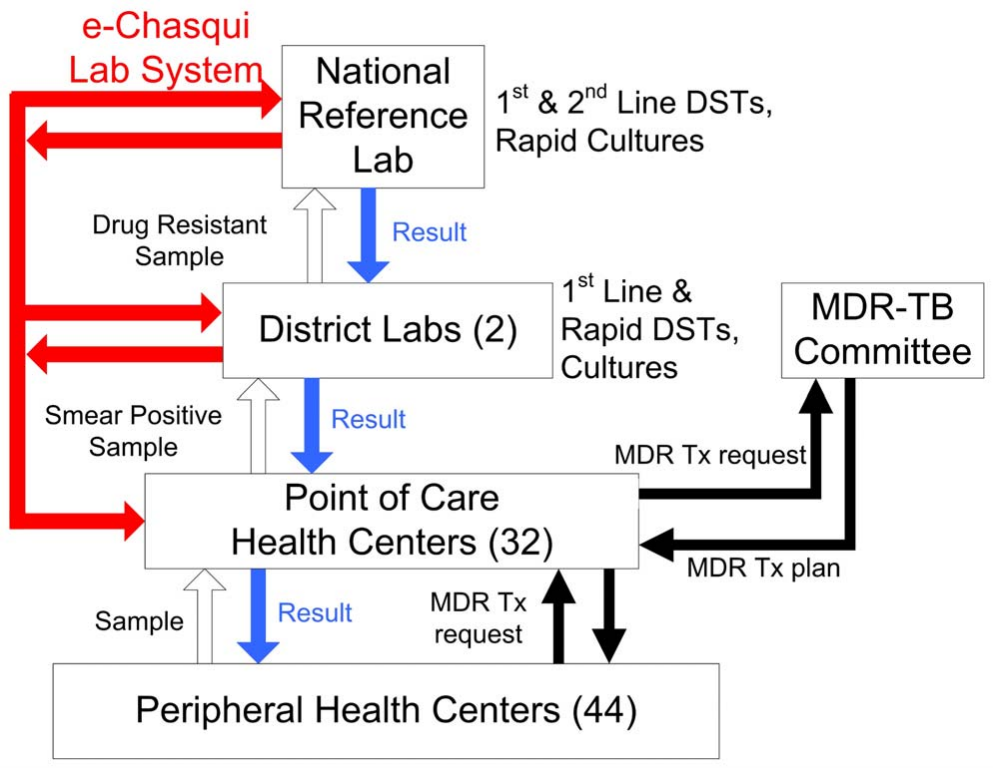
Flow of samples, results, and MDR treatment requests and plans within the Peruvian National TB Program. TB microscopy is carried out at point of care health centers, and smear positive samples are only referred for DST if there are risk factors for MDR TB.

This study was carried out in two health districts of Lima, Peru: Lima Ciudad and Lima Este. Lima Ciudad has 45 health establishments (24 HCs, nine health posts, and 12 hospitals) serving a population of 1,577,090 in an area of approximately 100 km^2^. Lima Este has 134 health establishments (42 HCs, 87 health posts, and 5 hospitals) serving a population of 1,088,515 in an area of approximately 6340 km^2^. Smear microscopy is used to diagnose active TB, while culture and drug susceptibility testing (DST) are reserved for individuals with confirmed TB and at least one risk factor for MDR-TB according to National Tuberculosis Program (NTP) Norms [18]. Smear microscopy is performed in Level One laboratories in HCs and hospitals. Health Posts send sputum samples to their closest HC for smear microscopy. For patients with MDR-TB risk factors, smear-positive samples are sent to district laboratories for culture and/or DST to first-line drugs. These laboratories used the Ogawa method for cultures, and for DSTs either a direct nitrate reductase assay (NRA), also termed Griess, or the indirect conventional method using a culture sample. Strains resistant to isoniazid and/or rifampicin are sent to the NRL for DST to second-line drugs using the indirect conventional method. Paper results are generated by the NRL and district laboratories and transported to health establishments. The patient is then routinely seen by a pulmonologist at the local hospital to review the DST results and, if necessary, modify the TB regimen. In new patients with drug-resistant isolates, a district expert committee must review the case and approve initiation of MDR-TB therapy (Figure 1).

The two health districts organize transmission of paper results to HCs differently. In Lima Ciudad, all 24 HCs are point of care HCs that receive results directly from the district laboratory. In Lima Este, health establishments are organized in “micro-networks.” Seventeen point of care HCs serve as the micro-network heads, and use the identical process as HCs of Lima Ciudad. The other 25 HCs and 87 health posts are peripheral HCs, which receive test results via the head of its micro-network (Figure 1). In the intervention group, point of care HCs had internet and therefore direct access to eChasqui. These HCs could print the results from e-Chasqui to send to the peripheral HCs or wait for the paper copy to arrive from the lab to send on.

### Randomization and Masking

In March, 2006, e-Chasqui was first implemented at the two district laboratories and the NRL. These laboratories served all of the health establishments. After full implementation in the laboratories, HCs were randomly assigned to have access to e-Chasqui versus no access following simple randomization procedures to intervention or control groups, with no allocation concealment. In Lima Ciudad, the 20 highest TB incidence HCs were randomly assigned, six to e-Chasqui and 14 to controls. Only six were assigned to e-Chasqui due to the limited implementation resources. In Lima Este, the 12 micro-networks within Lima city limits were randomly assigned, six to e-Chasqui and six to control (Figure 1). The six micro-networks assigned to e-Chasqui consisted of six point of care HCs with 17 peripheral HCs; the six control micro-networks were comprised of 6 point of care HCs with 27 peripheral HCs.

### Study Design

We performed intent-to-treat analysis based on the original HC randomization. During the study (October 2006), the Lima Este health district re-organized their micro-networks which had the following impact on study assignments: one point of care control HC became a peripheral intervention HC; three peripheral intervention HCs became peripheral control HCs; and three peripheral control HCs became peripheral intervention HCs. Data from these “cross-over” HCs only affected 17 data points for the primary outcome and would be expected to dilute the effect of the intervention.

### Study Population

All individuals who lived within the catchment area of participating health centers and had at least one MDR-TB risk factor as defined by the Peruvian NTP Norms were included in this study [18]. There were no exclusion criteria for enrollment into the study. Eligible subjects were identified when sputum samples were submitted to the district laboratory for DST, and eligibility criteria were confirmed by chart review.

### Outcomes

The primary outcome of the study was the laboratory turn-around-time (TAT), defined as the number of days between a test result date and the date that result was received by the treating HC (Table 1). This is different from some studies that define laboratory TAT from the date the sample was collected or sent to the laboratory. For the paper system, the date received at the HC was the reception of the paper result as shown in the reception stamp by the HC. For the electronic system, the date received at the HC was the earliest receipt of either the paper result or electronic result (i.e. date viewed online by a TB staff member which was logged in the system). This primary outcome was calculated for both cultures and DSTs. Secondary outcomes can be seen in Table 1.

**Table 1:** Outcome definitions for turn-around-times (TAT) and their respective sample.

Outcome	Definition	Sample
Culture Lab TAT	Number of days between a culture result date and the date that result was received by the HC	All cultures performed on participants belonging to study clusters
DST lab TAT	Number of days between a DST result date and the date that result was received by the HC	All DSTs performed on participants belonging to study clusters
DST lab TAT >60 days	The proportion of DST results with a laboratory TAT greater than or equal to 60 days	All DSTs performed on participants belonging to study clusters
Treatment TAT	Number of days between the result date of the first DST resistant to INH, RIF, or both and the date of a starting a second line regimen for the first time, or if a patient was on second line treatment, the removal of a medication to which the patient was resistant, or the addition of a medication to which patient was susceptible.	All patients with a DST resistant to INH, RIF, or both
Culture conversion TAT	Number of days between the result date of the first DST resistant to INH, RIF, or both and the sample date of the first of two negative consecutive cultures taken at least 30 days apart	All patients with a DST resistant to INH, RIF, or both who had a positive culture six month before or two months after the DST result date

### Intervention

We designed and implemented the web-based laboratory information system “e-Chasqui” to improve the timeliness and quality of laboratory data [15]. It was deployed in the NRL, two district laboratories, and 12 intervention HCs. The core of the e-Chasqui interface is a single patient page containing the history of all tests performed for the patient on a left sidebar, and the details for any single sample on the main part of the page. Tools built for the laboratory include quality control, reports on tests performed, warnings for delayed reporting of results, and a user directory to control any individual's access. Tools for the laboratory were used for both intervention and control samples. Tools for clinicians (in intervention sites) included email notification of new results, a consolidated list of results for their jurisdiction, and a list to track the status of all pending samples. All intervention HC staff were trained at their HC in an initial session for approximately one hour. The data administrator would then visit or call the HC at least twice a month and could be contacted via cell phone or email during business hours.

e-Chasqui was built as a stand-alone module on the open source Partners In Health Electronic Medical Record (PIH-EMR), a web-based system designed for TB and MDR-TB treatment in resource-poor settings [19]. The PIH-EMR provides the ability to register patients, order medications [20], display chest x-rays, generate monthly reports for funders, and predict future drug requirements [21]. This system has been adopted as the official system of the Peruvian NTP.

### Sample Size

Previously we measured the treatment TAT to be approximately 65 days [14]. Assuming that the effect estimate of e-Chasqui would reduce this delay by 20 days, based on 0.8 power, and a two-sided alpha of 0.05, 165 subjects in each group (330 total) were required.

The sample population for the primary outcome (laboratory TAT) was all samples sent to the participating laboratories. The analysis for treatment TAT was limited to patients with a DST showing MDR-TB and the analysis for culture conversion TAT was limited to patients with a DST showing MDR-TB that converted their cultures.

### Data Abstraction

Baseline data were collected 15 months prior to the implementation of e-Chasqui (Jan 1, 2005–Mar. 30, 2006 for Lima Ciudad, May 1, 2005–Aug. 18, 2006 for Lima Este). However, the Lima Este district laboratory did not perform DST before the implementation of e-Chasqui, hence there are no pre-implementation data on DSTs for that district.

Data were prospectively abstracted by a team of trained collectors who used standardized forms. For the RCT, the study started on the date of implementation of e-Chasqui and ended on August 31, 2008. We also used data from the e-Chasqui database, including the date of electronic receipt and viewing of test result. If the end date for any TAT was missing, we censored that time using the date the patient finished the study.

### Statistical Analysis

We examined the effect of the intervention at a sample and patient level, adjusting for the impact on variance of the clustering in the study design. We used multivariate regression models (marginal model with generalized estimating equations) to investigate the effect of the intervention on the TAT outcomes as a function of covariates and to account for the clustering at the HC level [22]. To investigate whether the intervention was associated with a reduction in the number of DST results with laboratory TAT greater than 60 days, we used a generalized linear mixed model [23] with HC as a random effect and health district and period (pre- and post-implementation) as fixed effects.

To adjust for possible HC differences that may have been unequally distributed despite randomization, we included the median pre-intervention TAT per HC for each of the TAT outcomes (as a proxy for HC variance) and number of HC staff changes. At the individual level for the treatment and culture conversion TAT, we also adjusted for HIV and pediatric status. We used SAS version 9.1 (SAS Institute, Cary, NC, USA) [24] for all analysis and checked all models built using R [25].

### Data Sharing

Data sharing: statistical code and dataset available from the corresponding author at mailto:h.fraser@leeds.ac.uk. Patient consent was not obtained but the presented data are anonymised and risk of identification is low.

## Results

**Figure 2:**
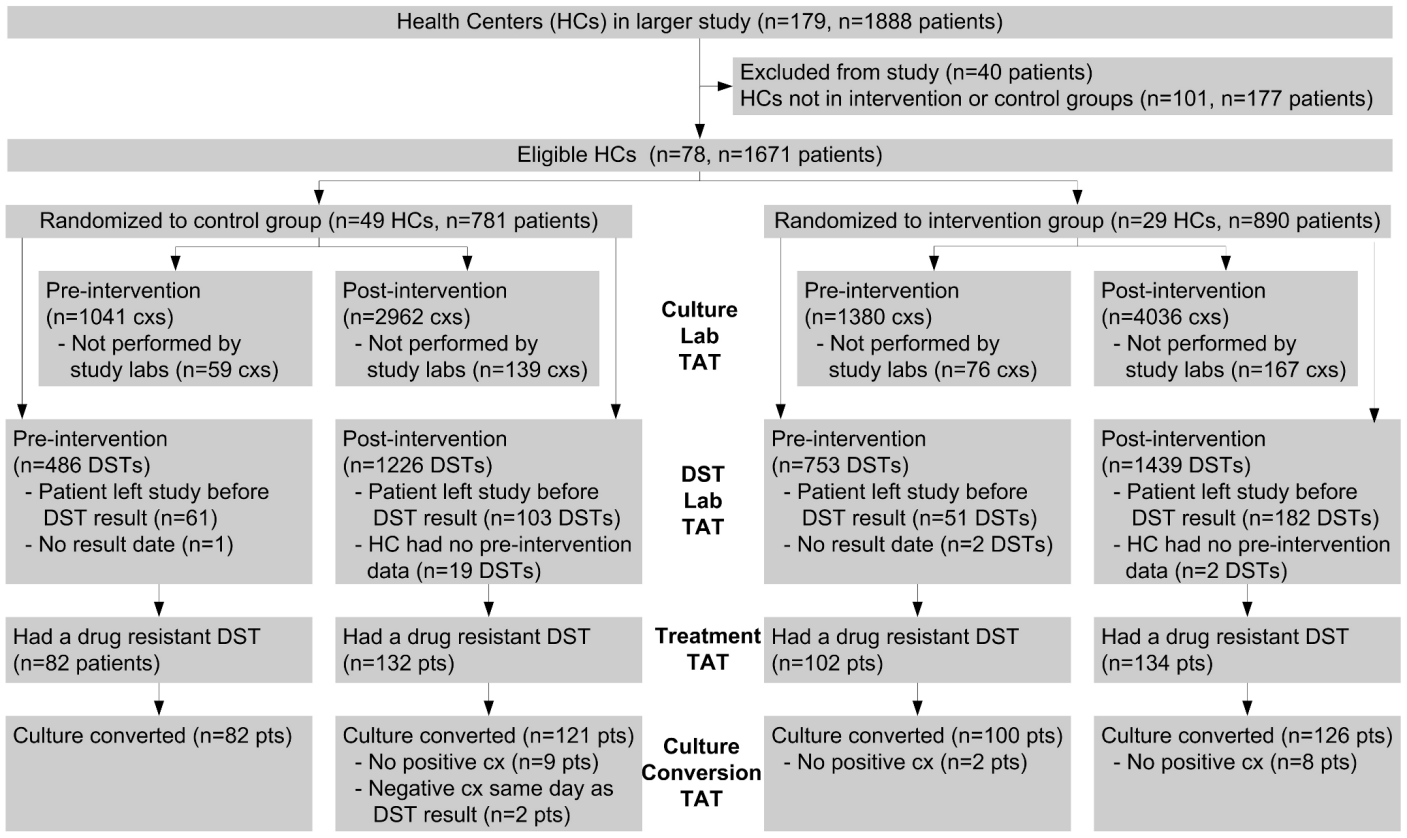
Flow of participants, cultures and DSTs through trial. The Pre-intervention groups represent baseline data collection prior to the RCT which was used to correct for baseline differences between sites during the analysis. Cx is sputum culture, DST is drug sensitivity test.

During the trial, 89% (1671/1888) of all eligible patients were enrolled (Figure 2). The intervention HCs had a significantly greater number of study participants per HC. If separated by district, these differences existed only in Lima Ciudad. The intervention HCs also had younger participants, more female participants, and fewer numbers of personnel changes in TB clinicians per HC. There were no significant differences in the number of patients per peripheral HCs; number co-infected with HIV; turnover of TB nurses during the study; number of patients with baseline positive smear or culture status or with drug-resistant TB by study arm (Table 2). 98% of all culture results and 100% of all DST results available in e-Chasqui were viewed by the intervention HCs.

**Table 2:** Characteristics, outcome measures, and sample sizes for all study health centers (HCs) and participants.

Characteristic	Control	Intervention	p-value
Total # point of care HC	20	12	
Total # peripheral HCs	27	17	
Total # (%) participants in Lima Ciudad	356 (46)	463 (52)	
- Total # (%) participants in point of care HCs	547 (70)	651 (73)	
- Total # (%) participants in peripheral HC	233 (30)	240 (27)	
Participants per HC	16.3 (16.9)	29.7 (32.6)	0.04
Participants per point of care HC	26.1 (18.2)	54.2 (39.1)	0.02
Participants per peripheral HC	8.6 (10.9)	13.3 (10.5)	0.10
Smear or culture positive patients per HC	12.8 (13.7)	23.7 (29.5)	0.08
Patients with drug resistant DST per HC	4.9 (4.8)	8.3 (11.7)	0.73
Age (years)	33.5 (16.0)	31.1 (16.5)	0.001
Total # (%) female	257 (33)	340 (38)	0.02
Total # (%) co-infected with HIV	100 (13)	94 (11)	0.15
Changes in TB clinician per HC during study	2.1 (1.1)	1.7 (1.1)	0.04
Changes in TB nurse per HC during study	1.6 (0.9)	1.4 (0.9)	0.34
**Sample Sizes**			
	Control	Intervention	
Cultures Sampled	3101	4203	
DSTs Sampled	1348	1623	
	Before e-Chasqui	After e-Chasqui	
Cultures Sampled	1100	1457	
DSTs Sampled	547	804	

For characteristics, values are mean (SD) unless stated otherwise. The sample sizes are shown for both the control/intervention, as well as for before and after the implementation of e-Chasqui.

### TAT Outcomes

Intervention HCs took significantly less time to receive both DST (median 11 vs. 17 days, p<0.001) and culture (5 vs. 8 days, p<0.001) results (Table 3). Intervention HCs had 47% fewer DSTs with a laboratory TAT of greater than 60 days compared to control HCs, however this was not significant (p = 0.12). For a total of 266 participants (134 in the intervention HCs, 132 in the control) treatment TAT did not significantly differ in the intervention versus control HCs (median 88 v. 77 days, p = 0.28).

**Table 3:** Primary and secondary outcomes.

Outcome	Control HCs	Intervention HCs	Adjusted Hazard Ratio (95% CI)	p-value
Culture laboratory TAT	8 (7)	5 (5)	0.68 (0.65–0.72)	<0.001
DST laboratory TAT	17 (24)	11 (19)	0.67 (0.62–0.72)	<0.001
DST laboratory TAT >60 days	21.8 (294)	11.6 (188)	0.63 (0.35–1.13)	0.12
Treatment TAT	77 (91)	88 (97)	0.82 (0.55–1.22)	0.28
Culture conversion TAT	79 (69)	63 (85)	0.72 (0.51–1.00)	0.047

Figures are median number of days and IQR in parentheses except for DST laboratory TAT >60 days, which is percentage and absolute value in parentheses.

Among 247 participants included in analysis for culture conversion TAT (126 in intervention HCs, 121 in control), those in the intervention HCs achieved culture conversion 16 days sooner, 20% less than those in the control HCs (p = 0.047, Table 3).

## Discussion

The e-Chasqui laboratory information system considerably reduced the time to communicate results of cultures and DST to local HCs and the proportion of results that had an excessive delay or never arrived, though this last value was not statistically significant. Although intervention versus control groups did not significantly differ in time to treatment, those in the e-Chasqui group did have a significant decrease in the time to culture conversion, compared with the control group.

The results reported here show that information systems can have a large effect in the communication time of critical laboratory data. This effect might be even greater if this system were used in rural areas since the obstacles to communication usually encountered (long travel times, infrequent transport, or weather) are easily surpassed with an internet connection (fixed or cellular). Further, this system can prevent patients from “falling through the cracks” since without an integrated electronic system, it is difficult to identify how many confirmed TB patients are not on treatment. This “break” in the patient care process can be easily overlooked when evaluating a TB program but is a critical issue in the effective scale up of treatment to hundreds or thousands of patients.

A previous report from this study compared the effect of the eChasqui system on delays in receiving results at health center that have the system accessible on the clinician's desks tops “point of care” and those that receive results printed at a nearby direct health center “peripheral.” [26] This showed that the benefits in reduction in delays are only seen in sites where the clinician directly accesses the electronic system. It also showed that in point of care health centers there was a significant reduction in the proportion of DST results with laboratory TAT greater than 60 days (p<0.001).

An important finding was that the patients in the intervention HCs achieved culture conversion 16 days earlier than those in the control HCs, a 20% decrease. The mechanism of this impact, however, is unclear since it was not due to an earlier start of appropriate treatment (although the clinicians had access to the DST data earlier, the management of the start of treatment was through a committee and not part of the study protocol). We believe that the culture conversion TAT measures not only the effect of the drug regimen, but other factors not directly measured including: 1) significantly increasing the number of results received at the HC; [16] 2) improved monitoring of patients because clinicians have greater access to their history and laboratory data (clinicians did in fact send a mean of 4.7 cultures in intervention HCs versus 3.98 in control HCs) ; 3) increased ability to prioritize regimen changes; 4) email alerts to the TB staff leading to better patient monitoring; and 5) improved adherence by patients that believed they were receiving better treatment since their doctor is using a computer instead of paper, or is providing more attentive follow-up. Other factors that can effect time to culture conversion include medication adherence, resistance pattern, disease severity, and prior treatment failure. The intervention and control groups should be equivalent in these factors, although this was not documented in the study. Further studies are needed to clarify the impact of more rapid and reliable laboratory systems on time to culture conversion.

A recent systematic review we carried out confirmed that there have been no prior randomized controlled trials showing an electronic health information system decreasing laboratory delays in a resource-poor setting [8]. The review was updated for this study. In addition few prospective, randomized trials worldwide have shown that any type of information system can have a clinical impact.

Another reflection of the system's success is its expansion by the Peruvian Ministry of Health to serve a network of 259 institutions serving a catchment area of over 4.1 million people and providing treatment to approximately 9,600 TB and 1,100 MDR-TB patients every year. Since the end of the study, the laboratories have taken over the data management and the Peruvian non-profit Socios en Salud Sucursal Peru provides technical support. The cost of running eChasqui for a year (in 2007) was calculated to be US$34,738 total or US$0.53 per sample analysed. This was equivalent to approximately 1% of the National TB program budget. The costing is described in detail in an earlier report [15].

There were fundamental baseline differences between the intervention and control HCs despite the randomized nature of this trial. These differences could introduce bias into the analysis, but we used pre-implementation values in our models to account for measurable confounders. The study was conducted in the two most populous health districts in Peru. Therefore the generalizability of these results should be treated with caution. Being in a semi- urban area provided the project with mostly consistent power and internet, as well as geographic proximity to provide technical support, which could be more limited in other settings. Finally, this was a formative, rather than summative, evaluation since the developers were involved.

This system only directly intervened in the first step of a patient's treatment which was the communication of results to clinical staff. We believe that the effect of a system could be increased by incorporating additional system tools, such as tools to create summaries of clinical and laboratory data for the committee recommending patients for MDR-TB treatment, computerized initiation and tracking of regimen changes [20] and tools to aid follow up of patients.

There is great potential to scale the use of information systems for sample tracking and laboratory reporting. Use of mobile phone based software (mHealth) has been shown to be less costly than paper [27] and have similar benefits as described here [28]. The rapid growth of mobile phone networks even in rural Africa is starting to have an impact on delivery of laboratory data as shown in a recent non randomized study of pediatric HIV care in Zambia [5]. In addition we have built similar tools to eChasqui in the open source EMR system OpenMRS, which is used in more than 50 developing countries (www.openmrs.org) and can be freely downloaded from the internet [29], [30]. A demonstration of OpenMRS adapted for MDR-TB is available at www.openmrs.org/demo.

## Conclusion

A carefully designed and implemented web-based tuberculosis laboratory information system reduced the time to communicate results between laboratories and health establishments spread throughout a large, peri-urban area. Patients in intervention and control HCs did not significantly differ in time to treatment, but time to culture conversion was 16 days faster, compared with those in control HCs (20% earlier). This system has been expanded to 259 health centers serving a population of 4.1 million. Although there are new genomic analysis tools including “GeneXpert™” [31] which detect TB infection and Rifampicin resistance within two hours, DST is still required to assess the full resistance pattern. All laboratory samples and data must be tracked to ensure the results reach the treating physicians especially in many smaller clinics. A system like eChasqui should be considered as a component of laboratory infrastructure to support TB and MDR-TB care in other resource-poor settings. It could also strengthen the large scale expansion of care for MDR-TB proposed by the WHO over the next 5 years, and assist with disease surveillance.

This is to our knowledge, the largest randomized controlled trial of a patient specific health information system to support clinicians in a developing country, and the first laboratory information system to show a clinical impact worldwide. This technology has great potential to scale up and to impact quality of care in many countries. While the focus here is on drug resistant TB, the results are relevant to other diseases, in particular CD4 counts and viral loads for HIV management [5], [32], [33], and access to histology results in oncology care.

## Supporting Information

Checklist S1 **CONSORT checklist.** List describing the location of the requirements of the CONSORT statement.
(PDF)

Protocol Secondary Use S1 **IRB Protocol Secondary Use.** Form used to receive IRB approval since this study was limited to the secondary use of research samples or data. Secondary use is the use of existing research samples and/or data for a new research project.
(PDF)

Procotol Summary S1 **IRB Procotol Summary.** Summary of the study protocol submitted to the Internal Review Board (IRB).
(DOC)
