# Supplementary material for: Reducing Communication Delays and Improving Quality of Care with a Tuberculosis Laboratory Information System in Resource Poor Environments: A Cluster Randomized Controlled Trial
Source: PLoS One. 2014 Apr 10;9(4):e90110. doi: 10.1371/journal.pone.0090110 (PMC3982951; doi:10.1371/journal.pone.0090110)
Supplement: Protocol Secondary Use S1 — IRB Protocol Secondary Use. Form used to receive IRB approval since this study was limited to the secondary use of research samples or data. Secondary use is the use of existing research samples and/or data for a new research project. (PDF) [file pone.0090110.s002.pdf]

SECONDARY USE OF RESEARCH SAMPLES/DATA

This form may be used for studies limited to the secondary use of research samples or data. ***Secondary use*** is the use of existing research samples and/or data for a new research project.

1. PRINCIPAL/OVERALL INVESTIGATOR: (cannot be resident or research fellow)

Name: **Sonya Shin, MD**
First Name, Middle Initial, Last Name, Degree(s)

Institution: ☒ BWH ☐ DFCI ☐ MGH ☐ SRH Employee ID#: **011 806 536**

Dept/Service: ______ Div/Unit: **Social Medicine and Health Inequalities**

Address: **1620 Tremont Street Boston, MA 02120**

Telephone: **(617) 732-6438** Beeper: ______ FAX: **(617) 432-5300**

E-Mail: **sshin@partners.org**

2. CO-INVESTIGATORS/STUDY STAFF: (list institution in parenthesis, if not BWH or MGH staff)

Hamish Fraser, MBChB, MSc, MRCP
Joaquin Blaya, M.S. (HMS)
Jaime Bayona, MD, MPH

Send copies of correspondence to:
Charmaine Lastimoso
Division of Social Medicine and Health Inequalities
Brigham and Women's Hospital
1620 Tremont Street, 3rd Floor
Boston, MA 02120-1613

3. STUDY TITLE

Operational assessment of laboratory information system for MDR-TB in Lima, Peru

4. FUNDING SOURCE: (if federally funded, e.g., NIH, DOD, etc., submit entire copy of grant with form)

MIT William Asbjornsen Albert Memorial Fellowship
Office of AIDS Research, National Institute of Health
Bill and Melinda Gates Foundation
Has this project been awarded/funded at the time of this submission? ☐ YES ☒ NO

5. PURPOSE AND DESCRIPTION OF THE STUDY

The objective of this study is to compare the effects of a web-based laboratory information system (e-Chasqui) between a network of health establishments with access to e-Chasqui (intervention group) compared with a network of health establishments without access to e-Chasqui (control group).

The specific aims are:

1. To compare the "laboratory turn-around-time" (from date a culture or drug susceptibility test (DST) result obtained to date result obtained at health center) of samples pertaining to health establishments in the intervention versus the control group.

2. To compare the "clinical turn-around-time" (from date DST result obtained to date patient

evaluated by physician with that result) among MDR-TB patients pertaining to health establishments in the intervention versus control group.
3. To compare the laboratory reporting errors (defined as incorrect smear, culture, or DST result) among health establishments in the intervention versus control group.
4. To qualitatively assess the acceptability, usability and factors in appropriation of e-Chasqui among users in health establishments with access to the system.

6. SAMPLES/DATA TO BE USED: (describe briefly)

We will use data that is routinely generated as part of patient care and is being collected as part of the overarching study. The data will include the smear, culture and DST results, processing times of data collection/entry and discrepancies in data comparing the e-Chasqui with the laboratory registers.

7. FETAL TISSUE

Will this study involve the secondary use of **fetal tissue**? ☐ YES ☒ NO

If **YES**, explain why fetal tissue, rather than other tissue, is required to achieve the research goals.

If **YES**, indicate original source of the fetal tissue:

The research use of fetal tissue is covered by Federal and State regulations (M.G.L. - Chapter 112, Section 12J). If the original source of the fetal tissue was a supplier of biological materials, the supplier must be in compliance with all applicable Federal and State regulations and laws.

8. SOURCE OF SAMPLES/DATA AND RELATED INFORMATION

8a. Where will you obtain the samples/data (check all that apply):

☐ Collaborators within Partners, specify:

☒ Collaborators at outside institutions, specify: **Socios en Salud (Partners in Health in Peru), Peruvian National Tuberculosis Program (NTP) and National Institute of Health (INS)**

☐ Other, specify:

8b. Do you plan to re-contact subjects? ☐ YES ☒ NO

If YES, do not complete this form – complete the standard application form.

8c. Will the research be limited to the use of existing samples/data? ☒ YES ☐ NO

8d. Do samples/data retain a code linking sample/data to individual human subjects? ☒ YES ☐ NO

If **YES**, will key to the code or identity of the subjects ever be known to you? ☒ YES ☐ NO

If **YES**, explain below why you need to identify the subjects:

This is data for current patients that is currently used by Socios en Salud and INS in clinical care of the patients. We are only implementing a new method of collecting this data.

8e. Will samples/data be sent to individuals or institutions outside Partners HealthCare? ☒ YES ☐ NO

If **YES**, what information will be sent with the samples/data?

All information is maintained within Socios en Salud/Partners in Health and the Peruvian National Institute of Health

NOTE: If the research involves sending human material or tissue to collaborators outside Partners, the material can be sent only after an appropriate agreement has been signed by Partners Corporate Sponsored

Research and Licensing on behalf of the investigator.

9. DATA TO BE USED

9a. Data to be used: (check all that apply)

- ☒ Personal data (name, address, PCP)
- ☐ Demographic data (age, gender, vital status)
- ☒ Laboratory data
- ☐ Images
- ☐ Other, please specify:
- ☐ Billing data
- ☐ Coded encounter data (diagnoses, procedures, dates)
- ☐ Reports, clinic/office notes

9b. Explain why the research could not be done without access to this data.

This study is to assess the benefits of implementing a new process of maintaining these lab results. Personal data is necessary to link laboratory data with subjects.

10. PRIVACY/CONFIDENTIALITY PROTECTIONS: (address use/disclosure of identifiable information)

Check below any of the following identifiers that will be recorded with or linked by code to the data:

Data that are coded, where the key to the code is accessible to researchers, are considered protected health information (PHI) subject to HIPAA regulations.

- ☒ Name
- ☐ Social security number
- ☒ Medical record number
- ☐ Address by street location
- ☐ Address by town/city/zip code
- ☐ Dates, e.g., date of birth; admission/discharge date; date of procedure; date of death
- ☐ Telephone number
- ☐ Fax number
- ☐ Electronic email address
- ☐ Web URLs
- ☐ Internet protocol (IP) address
- ☐ Health plan beneficiary number
- ☐ Account number
- ☐ Certificate/license number
- ☐ Vehicle Identification number and serial number, including license plate number
- ☐ Medical device identifiers and serial numbers
- ☐ Biometric identifiers (finger and voice prints)
- ☐ Full face photographic image
- ☐ Any other identifier likely to identify the subject

The following questions (10a.-10c.) must be addressed if any of the above identifiers are temporarily or permanently recorded with or linked to the data:

10a. How will the protected health information (PHI) be stored and protected?

<u>For paper-based information</u>, describe where the identifiable information will be stored, who has access to the storage area, and how that access will be audited. If the information is stored off-site, describe how security at the facility is maintained and whether or not a business associate agreement has been or will be signed. At a minimum, consider storing PHI in locked drawers, cabinets or offices with access restricted to the principal investigator (PI) and study staff designated by the PI. **<u>For electronic information</u>**, describe how electronic security is maintained, including what password protections and virus software are enabled. Describe how the system will be audited. At a minimum, consider storing PHI in a password-protected Partners computer with virus software.

Research staff is already trained in the importance of maintaining confidentiality, and all staff members will sign a certificate of confidentiality. Hard copies of research material will be stored in locked cabinets within SES offices guarded by 24-hour security personnel. Data and culture specimens will be maintained at the regional laboratories, the INS and the NTP health establishments for issues of patient care.
For electronic information, the system used in this study is built on extensive previous work on encryption and web security for financial transactions and medical records.
1. Users are required to have complex passwords and can access only the parts of the site they need
2. All logins and viewed pages are recorded and reviewed to ensure that no unauthorized access occurs
3. A centralized database allows the computer and data to be physically secure and backed up regularly
4. Encryption of data transfers is done with the Secure Sockets Layer (SSL) protocol.
Further, the study personnel have all signed confidentiality agreements and have been trained on the proper use of the electronic information and research data.

10b. What individuals/entities will have access to protected health information (PHI)?

Describe what members of the study staff (including their role in the study and qualifications) will have access to the subjects' PHI. If persons or entities outside of the study staff (beyond those required for legal, institutional or accreditation review) will have access to the PHI, please provide their names and the reason why they require access. **Note: All disclosures of identifiable health information to persons or entities outside Partners must be tracked in accordance with the Partners policy "Accounting of Disclosures". A Research Tracking Tool is available on the HRC website** http://healthcare.partners.org/phsirb.

Since this is routine clinical data already being gathered, no individual will gain additional access to data due to this protocol. Dr. Sonya Shin, PI, is an assistant professor at DSMHI and has been working in Peru for over 15 years. Dr. Hamish Fraser, co-investigator, is a trained cardiologist, assistant professor at the BWH Division of Social Medicine and Health Inequalities (DSMHI) and Directors of Informatics at Partners in Health. Dr. Jaime Bayona, co-investigator, is the executive director of Socios en Salud and a lecturer at the Harvard Medical School Dept. of Social Medicine. Mr. Joaquin Blaya, research coordinator and manager, is a PhD student at the Harvard Medical School-MIT Division of Health Sciences and Technology (HST).

10c. What will happen to the protected health information (PHI) at the conclusion of the study?

Will this data be destroyed at the end of the study? ☐ YES ☒ NO

If the answer above is **NO**, explain why the data must be retained, including in part whether the data is needed for a health or research purpose, legal or institutional requirement, or other reason. Be specific.

The data is used for the clinical treatment of patients. This data has been collected for the past 4 years and is an integral part of patient follow up.

11. RISKS TO SUBJECTS

What are the risks to subjects whose information is used in this research? Specifically address risk to privacy. Explain why these risks are no more than minimal.

There are no directly foreseeable risks or discomforts to the subjects caused by participation in the study, since the subjects will have no contact with the study team. Further, the reduction indirect risks such as data error or confidentiality are aims of this study and therefore will be closely monitored.

12. INFORMED CONSENT

Were the samples collected as part of an IRB-approved protocol, with the informed consent of subjects? ☐ YES ☒ NO

If **YES**, provide a copy of the IRB-approved consent form, if available, and describe below how the proposed use is consistent with the use outlined in the IRB-approved consent form:

These samples are collected as part of routine clinical care. As such, a waiver was obtained from the Harvard Medical School IRB committee.

If the proposed use of the samples/data is not consistent with secondary uses outlined in the IRB-approved consent form, request waiver of informed consent and authorization below.

13. REQUEST FOR WAIVER OF CONSENT AND AUTHORIZATION

13a. Explain why the research could not practicably be done if informed consent or authorization were required.

Seeking patients' informed consent before including them in the evaluation would not be practicable for this study. Patients who refuse to participate in this study may be more likely to have MDR-TB or other causes of stigma (e.g. household contact with MDR-TB, HIV). Most MDR-TB patients in Peru reside in Lima's poorest neighborhood, in illegal and unstable conditions. It would therefore be impossible to obtain informed consent from all patients whose inclusion in the evaluation is crucial. For the same reason, no attempt will be made to provide patients with additional information after the study is completed.

13b. Explain why subjects' rights and welfare will not be adversely affected by the waiver.

Participation in the evaluation will not adversely affect the rights and welfare of the subjects. Participation in the evaluation will be determined by the patient's risk of MDR-TB, and will have no impact on the diagnostic method or treatment that the patient receives during or after the evaluation period. The investigators will have no contact with the patient or physicians treating the patients. Patients in the evaluation will be treated no differently than they would were the evaluation not conducted.

13c. Describe any plans for providing the subjects with any research findings, if applicable. If none, so state.

There are no plans for directly providing the subects with any research findings, although results will be published in peer-reviewed journals and therefore accessible to the public.

14. WRITTEN ASSURANCE AND SIGNATURE

As Principal Investigator, my signature below provides written assurance that identifiable information will not be reused or disclosed except as required by law; for authorized oversight of the research project; or for other research only if that research has been reviewed and approved by the HRC/IRB with specific attention to and approval of the issue of access to this identifiable information.

Signature of Principal/Overall Investigator Date
