# Supplementary material for: Reducing Communication Delays and Improving Quality of Care with a Tuberculosis Laboratory Information System in Resource Poor Environments: A Cluster Randomized Controlled Trial
Source: PLoS One. 2014 Apr 10;9(4):e90110. doi: 10.1371/journal.pone.0090110 (PMC3982951; doi:10.1371/journal.pone.0090110)
Supplement: Procotol Summary S1 — IRB Procotol Summary. Summary of the study protocol submitted to the Internal Review Board (IRB). (DOC) [file pone.0090110.s003.doc]

# **IRB Procotol Summary S1**

# PRINCIPAL/OVERALL INVESTIGATOR

Sonya Shin

# PROTOCOL TITLE

Operational assessment of laboratory information system for MDR-TB in Lima, Peru

# FUNDING

MIT William Asbjornsen Albert Memorial Fellowship

Office of AIDS Research, National Institute of Health

Bill and Melinda Gates Foundation

# SPECIFIC AIMS

Concisely state the objectives of the study and the hypothesis being tested.

The objective of this study is to compare the effects of a web-based laboratory information system (e-Chasqui) between a network of health establishments with access to e-Chasqui (intervention group) and a network of health establishments without access to e-Chasqui (control group).

The specific aims are:

1. To compare the “laboratory turn-around-time” (from the date a culture or drug susceptibility test (DST) result is obtained to the date the result is obtained at the health center) of samples pertaining to health establishments in the intervention versus the control group.
2. To compare the “clinical turn-around-time” (from the date the DST result is obtained to the date the patient is evaluated by a physician in possession of that result) among MDR-TB patients pertaining to health establishments in the intervention versus control group.
3. To compare the laboratory reporting errors (defined as incorrect smear, culture, or DST results) between health establishments in the intervention versus control group.
4. To qualitatively assess the acceptability and usability of e-Chasqui among users in health establishments with access to the system.

We aim to test the following hypotheses:

1. The laboratory turn-around-time for health establishments with e-Chasqui access will be smaller than that for establishments without e-Chasqui access.
2. The clinical turn-around-time for patients pertaining to health establishments with e-Chasqui access will be smaller than that for patients in establishments without e-Chasqui access.
3. Health establishments with e-Chasqui access will have fewer errors compared to those without e-Chasqui access.
4. Factors associated with acceptability and usability of e-Chasqui by systems users can be identified.

# BACKGROUND AND SIGNIFICANCE

Provide a brief paragraph summarizing prior experience important for understanding the proposed study and procedures.

Delays in starting patients with multi-drug resistant tuberculosis (MDR-TB) on appropriate medication treatment lead to worsened patient outcomes [Telzak et. al, 1995] and increased risk of transmission. In Peru, the National Tuberculosis Program (NTP) has provided treatment for patients with MDR-TB since 1996, with cure rates ranging from 48% to 83% [Suarez 2004; Mitnick 2003]. Nonetheless, significant delays exist in the diagnosis of MDR cases and timely treatment initiation, in particular since MDR-TB treatment has been scaled to a national level.

Our preliminary work has found that the average turn-around-time (TAT) from the request of a DST to the prescription of appropriate treatment was 148 days (4.9 months) [unpublished data]. To address this delay, the NTP and the National Reference Laboratory (NRL) have jointly implemented the programmatic, decentralized use of two rapid DST methods: radiometric BACTEC-460 system and the Griess phenotypic assay method. The implementation of the rapid DST methods is planned as a component of efforts to scale up MDR-TB treatment to achieve nation-wide coverage. The NRL is currently overseeing the process of transferring DST about first-line drugs using the Griess method to seven regional laboratories which currently provide coverage for 90% of the national TB burden. In two of these regions (Lima Este and Lima Ciudad), an observational study “Operational Assessment of Diagnostic Methods for MDR-TB in Lima, Peru,” has been undertaken as a collaborative effort between the Centers for Disease Control and Prevention (CDC), Harvard University, and the Peruvian Ministry of Health, to assess the impact of these two methods in reducing the time to culture conversion among patients with MDR-TB.

Despite these advances, our preliminary work has highlighted the importance of other factors that contribute to the delays in timely diagnosis and management of MDR-TB cases. In fact, a closer assessment of the overall TAT found that there are two time intervals that contributed most heavily to this delay: the time to process a DST (75 days), and the time from DST result to patient re-evaluation (66 days). While the first delay could be addressed by efforts to implement rapid diagnostic methods at the decentralized (regional) level, benefits from such an intervention would be attenuated unless “downstream” factors contributing to TAT delay was also addressed.

For this second delay, an information system could be used to virtually eliminate the time to communicate results between the different institutions. As we have shown in previous studies, systems for managing medical data in the MDR-TB treatment program in Lima are often complex and slow with a significant risk of data errors [Choi et al 2004]. In 2001 we developed and deployed a web based medical record system, the PIH-EMR, to assist in the clinical management of these MDR-TB patients [Fraser et al, 2002]. This system includes a substantial set of data entry and analysis tools for laboratory results including sputum smears, cultures and DSTs. The PIH-EMR can also alert clinicians to abnormal laboratory results either when they login to the system, on specially formatted patient reports, or by email. Over the last six months the PIH-EMR has been modified to support the decentralization of DSTs in the regional laboratories. This laboratory component of the PIH-EMR, termed “e-Chasqui,” permits web-based entry of culture and DST results at all regional and central laboratories performing these tests. In addition, the system includes applications to assess quality control, generate aggregate reports, notify health centers of new results or contaminated samples, and track enrolled patients and the status of pending laboratory tests.

The NTP and the NRL have established a collaborative agreement to integrate e-Chasqui into the current management of patients at risk of MDR-TB. The implementation of e-Chasqui is being currently undertaken in two regions and, if effective, will then be expanded to provide a national network of all laboratories performing DST. It will be unfeasible for us to implement the information system in all health centers simultaneously because of the intensive initial demands on resources and training. Therefore, the implementation of the information system will occur in stages. For each region, necessary infrastructure and training to use the information system (including internet access, computers, and training on data security and patient confidentiality, and the use of email and the information system) will occur in a stepwise process, first involving approximately one half of the participating health establishments and then involving the remaining half.

Because of this staged nature of “roll-out,” there will be a period of time during which only half of the health establishments will be using the information systems. During this period, we will conduct **Part A** of our study, comparing our study endpoints among those health establishments with access to the information system (the intervention group), and those without access (at that time) to the information system, the (simultaneous control group). Given our logistical and financial constraints, which make simultaneous implementation of this programmatic intervention unfeasible, and given the programmatic agreement to eventually implement the information system in all health establishments, we do not feel that this strategy for implementation or this study design is unethical. Afterwards all health establishments will gain access to the information system. **Part B** of the study will compare the same endpoints among health establishments with initial access to the information system (intervention group) to those endpoints in the same health establishments before they gained access to the information system (historical control group). This study is therefore a prospective observational study designed to measure the impact of this programmatic intervention.

The operational assessment of this informatics intervention will be essential to determine if the system is effective in reducing delays in the management of suspected MDR cases and therefore, whether the system should be implemented at the national level. It will assess the impact of e-Chasqui on reducing reporting delays and laboratory data errors, and improving patient outcomes. More broadly, this study would provide data on a critical research question in medical informatics: whether the use of information systems can be shown to improve quality of patient care. Few studies have been able to convincingly demonstrate such benefits in the US and virtually none in developing countries. By using the data being collected by the overarching study “Operational Assessment of Diagnostic Methods for MDR-TB in Lima, Peru,” this proposal offers a unique opportunity to assess the clinical effects of improving information flow.

# RECRUITMENT PROCEDURES

Explain in detail the specific methodology that will be used to recruit subjects at the Partners performance sites, specifically how, when, where and by whom subjects are identified and approached about participation. Provide details of remuneration, if applicable. Include any specific recruitment methods used to enhance recruitment of women and minorities.

The population for this study will be that of the overarching study. Their study population includes those individuals with pulmonary TB who have a risk factor for MDR-TB in the region of Lima Ciudad. Risk groups for MDR-TB are defined by the NTP norms for TB management, and are described in the overarching study protocol. The enrollment procedures for the study are described below.

The treating physician at the health establishment is the primary care giver for all patients with suspected or confirmed tuberculosis. At the time of TB diagnosis or during treatment, the treatment physician may identify a risk factor for MDR-TB. In such cases, a sputum sample is sent to the regional laboratory for DST, using a rapid method (a colorimetric nitrate reductase assay termed the Griess) to identify resistance to isoniazid and rifampicin.

Because all sputum samples of patients who have at least one MDR-TB risk factor will be sent to the regional laboratory for DST, subjects eligible for the study will be identified by this referral. The regional laboratory will contact our study team when an eligible subject has been identified; in addition, the study team will visit each regional laboratory on a weekly basis to review sample referrals and confirm that all eligible subjects have been identified.

Faulty enrollment through this method could occur through two means. First, the treating physician may incorrectly solicit a DST in a patient without an MDR risk factor; second, the care provider may fail to identify a patient with a MDR risk factor. For this reason, the study team will make weekly visits to each health establishment in the study region to review patient medical records. They will 1) confirm that all enrolled subjects have an appropriate MDR-TB risk factor, and 2) identify any subjects who are eligible for the study (i.e. have an MDR-TB risk factor) and have not been previously captured. If any such “missed” subjects are identified, the patient’s status will be discussed with the health establishment to confirm that the patient is being managed as per NTP norms. If the patient has received less than two weeks of TB treatment since identification of one of the above risk factors, then the patient will still be eligible for inclusion into the study.

Since this an observational study of a programmatic intervention, there will be no contact between study investigators and subjects. Therefore, subjects will not be recruited for participation in the study.

# CONSENT PROCEDURES

Explain in detail how, where and by whom consent is obtained and the timing of consent, i.e., how long subjects will be given to consider participation. In general, informed consent is to be obtained by a licensed physician investigator if outside the research realm consent would be obtained usually by a licensed physician. If subjects are unable to give consent due to age (minors) or current physical/mental condition, indicate from whom consent will be obtained, e.g., parent(s), legally authorized representative, next-of-kin, etc. If subject is unable to give consent, describe how subject's ability to consent will be assessed during the trial and how consent will be obtained in the event the subject regains ability to give own consent.

The consent procedures for the overarching study “Operational assessment of diagnostic methods for MDR-TB in Lima, Peru” are described below.

Informed consent will not be sought for data collection, for the following reasons:

1) Patients will not be put at risk by their participation in the study. The data collection process will require no contact with the patients, since the information will be abstracted from patient charts and lab records, and will only include information that is routinely collected in the course of providing clinical care to patients. Moreover, the treatment regimens and management strategies for patients will be determined by the standards of care in vigor in Peru at the time.

2) Participation in the evaluation will not adversely affect the rights and welfare of the subjects. Participation in the evaluation will be determined by the patient's risk of MDR-TB, and will have no impact on the diagnostic method or treatment that the patient receives during or after the evaluation period. The investigators will have no contact with the physicians treating the patients. Patients in the evaluation will be treated no differently than they would were the evaluation not conducted.

3) Seeking patients' informed consent before including them in the evaluation would not be practical for this study. Patients who refuse to participate in this study may be more likely to have MDR-TB or other causes of stigma (e.g. household contact with MDR-TB, HIV). Most MDR-TB patients in Peru reside in illegal and unstable conditions. It would therefore be impossible to obtain informed consent from all patients whose inclusion in the evaluation is crucial. For the same reason, no attempt will be made to provide patients with additional information after the study is completed.

4) Patient confidentiality will be maintained (see Privacy protection below).

# RESEARCH DESIGN AND METHODS

For studies involving treatment or diagnosis, indicate how the study procedures differ from standard clinical care or diagnostic techniques for the disease or condition being studied and provide information on alternative treatments or methods of diagnosis. Describe study endpoints. For studies where there is an available treatment modality, methods for ensuring the safety of participants should be explicitly described. This is especially true for studies involving use of placebo. Objective criteria for determining treatment failure are preferred.

This will be a prospective observational controlled study to compare the effects of a web-based laboratory information system (e-Chasqui) between a network of health establishments with e-Chasqui access (intervention group) compared with a network of health establishments without e-Chasqui access (control group).

The study is planned in two parts: **Part A** will be a simultaneous control study occurring in the “transition” period comparing endpoints in those health establishments with e-Chasqui access to those in matched health establishments without e-Chasqui access. **Part B** will be an historical control study comparing endpoints in the establishments which gain initial e-Chasqui access, before (historical control) and after (intervention) the implementation. (See study schema).

For **Part A** of the study, with the input from the local institutions, intervention and control health establishments will be matched by 1) baseline turn-around-time, and 2) number of MDR-TB patients to allow a rigorous evaluation of the effects of the intervention. While randomization of health establishments could theoretically address the possibility of unequal distribution of potential confounding differences among health establishments, the effectiveness of randomization is limited by the low total number of health establishments (22 health establishments). Therefore, we have chosen to match health establishments based on characteristics most likely to contribute to confounding differences in the analyses of our primary endpoints.

We have designed the study with both historical and simultaneous controls for the following reason. Although health establishments will be matched to reduce confounding differences in the control and intervention groups of Part A of the study, we cannot fully eliminate the possibility that differences in health establishments could confound the association between the intervention and the primary study endpoints. Therefore, **Part B** of the study serves as a secondary means of comparing the intervention versus non-intervention group, with health establishments serving as their own historical controls. We opted again Part A as the primary study design, because of the theoretical influence of potential calendar bias.

The total number of subjects to be enrolled study-wide is 1600.

**Inclusion and exclusion criteria are:**

Subjects may be included into the study if they have one of the MDR-TB risk factors determined by NTP norms (see overarching study protocol) and live in one of the two regions designated as study sites. There only exclusion criteria for enrollment into the study is if the person does not speak Spanish.

# Study procedures

# Study procedures will not differ from standard clinical care. The study procedures involve collection of data that is routinely collected for clinical purposes. The data collection procedures performed for the overarching study are as follows:

A study team – comprised of eight data collectors, two data entry personnel, and a study supervisor – will be trained in the collection and entry of data using standardized data intake forms. (See Appendices for data collection forms).

In addition to the data collected on the intake forms, data (culture and DST results, as well as dates of reception, processing and results) will be collected from the NRL and intermediate laboratories from the information system database. In addition data on costs for all direct services listed will be collected.

Data sources include: patient charts, interview with health providers to confirm or clarify accuracy of data in patient charts, microbiology registries at local, regional and national laboratories, and the information system database.

Upon identification of a study subject, as previously mentioned, a study team member will review the patient chart and initial laboratory data to confirm that the subject does meet eligibility criteria. This initial health establishment visit will also involve collection of baseline data.

For subsequent prospective data collection, a data collector will perform a monthly visit to each health establishment to collect ongoing information on service utilization, regimen changes, bacteriology data, and treatment outcomes. Again, interviews with health providers will permit clarification and identification of any missing data.

All forms will be checked for accuracy at the time of data entry. In addition, random sampling of 10% of all data collection forms will be checked against original data sources.

Data will be double entered into an Epi-info (CDC Foundation, Atlanta GA) database. No identifiable patient data will be entered into the database.

# Study endpoints

The following primary endpoints of this study are:

1. Median “Laboratory turn-around-time” (from the date a culture or drug susceptibility test (DST) result is obtained to the date when the result is obtained at health center).
2. Median “Clinical turn-around-time” (from the date the DST result is obtained to the date the patient is evaluated by a physician).
3. Percent laboratory errors, by type (see Appendices: Quality Control Form) of error for health establishments in the intervention versus the control group.
4. Qualitative assessment of the acceptability and usability of e-Chasqui among users in health establishments with access with to the system.

Secondary endpoints of this study will include

1. Reduction of patients with delay above 100 days between the date an MDR result was obtained to the treatment start date of an MDR-TB regimen
2. Reduction of patients with delay above 100 days between the date an MDR result was obtained and the date of change to an appropriate regimen based on the DST result [would only use MDR results for these endpoints because we have more possibility to intervene]
3. Reduction of time to resend a sample when the first sample is found to be contaminated (from date of test result found to be contaminated to date of request of second sample)
4. Approximate cost of the intervention as a whole and per patient

The cost effectiveness analysis of this information system will be included in a larger cost effectiveness study planned for the overarching study, “Operational Assessment of Diagnostic Methods for MDR-TB in Lima, Peru.”

#

# Ensuring the safety of participants

The information system will be implemented under program conditions and not as a study intervention. The study itself will involve the collection of de-identified data that are routinely recorded as part of patient care. Therefore, there are no safety risks to the subjects incurred by participating in this study.

# EQUITABLE SELECTION OF SUBJECTS

Explain the rationale for the involvement or exclusion of special classes of subjects, such as fetuses, pregnant women, children, prisoners, institutionalized individuals, or other who may be considered vulnerable populations, e.g., the economically or educationally disadvantaged, hospital employees, medical students, or patients from medical practices of the investigator(s). Address whether any one group will bear a disproportionate share of the burdens of research or, whether the benefits, to the extent anticipated, will be distributed fairly. Specifically address the enrollment of women and minorities, including subjects who do not understand English.

Patients to be enrolled include women, children, and pregnant women. The morbidity and mortality risk of untreated MDR-TB for all vulnerable groups is considered far greater than that of MDR-TB therapy. In addition, approximately 40% of the study population lives in poverty. All groups within this study population will bear the same burden of research and benefit equally from this research. All patients are Spanish-speaking.

# PRIVACY AND CONFIDENTIALITY

Describe methods used to protect the privacy of subjects and maintain confidentiality. In most research, assuring privacy and confidentiality is only a matter of following some routine practices, e.g., substituting codes for identifiers, removing face sheets (containing such items as names and addresses) from survey instruments containing data, properly disposing of computer sheets and other papers, limiting access to identified data, impressing upon the research staff the importance of confidentiality, and storing research records in locked cabinets. Additional measures, such as obtaining a Certificate of Confidentiality, may be needed if the research involves sensitive matters such as sexual behavior or criminal or illegal behavior. Specifically address where individually identifiable information will be stored and who will have access to personally identifiable information. If information on subjects, biological samples or individual test results will be sent to individuals outside Partners, indicate what information will be sent, to whom it will be sent, and how confidentiality will be maintained. Indicate whether any data and/or specimens will be maintained at Partners or non-Partners sites for future uses not described in the protocol.

The information system used in this study is built on extensive previous work on encryption and web security for financial transactions and medical records.

1. Users are required to have complex passwords and can access only the parts of the site they need.
2. Users will be given different access privileges according to their roles. All logins and viewed pages are recorded and reviewed to ensure that no unauthorized access occurs.
3. A centralized database allows the computer and data to be physically secure and backed up regularly.
4. Encryption of data transfers is done with the Secure Sockets Layer (SSL) protocol.

For the study of the information system, privacy and confidentiality will be ensured by substituting codes for identifiers, removing face sheets from survey instruments containing data, properly disposing of computer sheets and other papers containing confidential information, and limiting access to identified data. Study workers will need an individual computer code to access patient data. Research staff is already trained in the importance of maintaining confidentiality, and all staff members will sign a certificate of confidentiality. Hard copies of research material will be stored in locked cabinets. Data and culture specimens will be maintained at the regional laboratories, the NRL and the NTP health establishments for issues of patient care. Data needed for this study will be derived from these clinical databases, without any patient identifiers (codes will be substituted), and stored at the Center for Diseases Control and Prevention (CDC) and the Division of Social Medicine and Health Inequalities at the Brigham and Women's Hospital for research purposes. Again, this information will be maintained confidential in both sites even after the current protocol has been completed. No data or specimens will be maintained at Partners or non-Partners sites for future uses not described in this protocol.

# USE OF SPECIMENS/DATA FROM OUTSIDE INSTITUTIONS/INDIVIDUALS

If human material, i.e., samples or specimens, or data from non-Partners sites will be used in this research, indicate whether the samples or data will contain identifiers that could be used to link the sample or data to individual subjects. Indicate where and how the samples or data will be obtained and how they will be labeled. If another IRB has reviewed this research, attach a copy of the institution's IRB approval and IRB-approved consent form, if applicable, with the submission.

All data will be collected from the study site in Lima, Peru. No human materials will be collected from this site for study purposes (just the results of analyses performed on human materials for clinical purposes will be collected). The treatment program is not a Partners site, although Harvard Medical School and Brigham and Women’s Hospital are involved with the program. Exception from IRB approval at the Peruvian National Institute of Health (INS) will be sought because of the programmatic nature of this study. The INS approval of this petition will be forwarded once confirmed.
